# Enhanced glaucomatous damage accompanied by glial response in a new multifactorial mouse model

**DOI:** 10.3389/fimmu.2022.1017076

**Published:** 2023-01-17

**Authors:** Sabrina Reinehr, Renée M. Girbig, Kim K. Schulte, Janine Theile, M. Ali Asaad, Rudolf Fuchshofer, H. Burkhard Dick, Stephanie C. Joachim

**Affiliations:** ^1^ Experimental Eye Research Institute, University Eye Hospital, Ruhr-University Bochum, Bochum, Germany; ^2^ Institute of Human Anatomy and Embryology, University Regensburg, Regensburg, Germany

**Keywords:** autoimmune glaucoma, combination, intraocular pressure, glia, myelin, microglia, optic nerve, retinal ganglion cells

## Abstract

**Introduction:**

Glaucoma is a complex, multifactorial neurodegenerative disease, which can lead to blindness if left untreated. It seems that, among others, immune processes, elevated intraocular pressure (IOP), or a combination of these factors are responsible for glaucomatous damage. Here, we combined two glaucoma models to examine if a combination of risk factors (IOP and immune response) results in a more severe damage of retinal ganglion cells (RGCs) and the optic nerves as well as an additional glia activation.

**Methods:**

Six-week-old wildtype (WT+ONA) and βB1-Connective Tissue Growth Factor (CTGF) mice (CTGF+ONA) were immunized with 1 mg ONA (optic nerve antigen). A WT and a CTGF control group (CTGF) received sodium chloride instead. IOP was measured before and every two weeks after immunization. After six weeks, electroretinogram (ERG) measurements were performed. Then, retinae and optic nerves were processed for (immuno-) histology. Further, mRNA levels of corresponding genes in optic nerve and retina were analyzed *via* RT-qPCR.

**Results:**

Six weeks after immunization, the IOP in CTGF and CTGF+ONA mice was increased. The optic nerve of CTGF+ONA animals displayed the most severe cell inflammation, demyelination, and macroglia activation. Fewer numbers of oligodendrocytes were only observed in WT+ONA optic nerves, while more apoptotic cells triggered by the extrinsic pathway could be revealed in all three glaucoma groups. The number of microglia/macrophages was not altered within the optic nerves of all groups. The loss of neuronal cells, especially RGCs was most pronounced in CTGF+ONA retinae in the central part and this was accompanied by an enhanced activation of microglia/macrophages. Also, Müller cell activation could be noted in CTGF and CTGF+ONA retinae.

**Discussion:**

In this new model, an additive degeneration could be noted in optic nerves as well as in the number of RGCs. These results suggest a potential additive role of high IOP and immune factors in glaucoma development, which will aid for understanding this multifactorial disease more precisely in the future.

## 1 Introduction

Globally, glaucoma is the second leading cause of blindness after cataracts (1). This optic neuropathy is characterized by a loss of retinal ganglion cells (RGCs) accompanied by excavation and degeneration of the optic nerve (2–4) and can be difficult to diagnose, particularly early in its clinical course. This is because patients only at an advanced disease stage notice visual field defects, when already about 30% of the RGCs are lost (5–7). In 2040, estimated 111 million people will be affected by glaucoma, many of them will be bilaterally blind (8–10). The exact pathomechanisms of this disease are not fully understood yet, however, an increased intraocular pressure (IOP) is related to RGC death and is considered the main risk factor (3, 11, 12). Additionally, an elevated IOP is associated with a higher prevalence (13–15) and incidence (16–19) of primary open-angle glaucoma (POAG), the most common form. However, approximately 30% of patients develop typical damage independent of IOP. This form is called normal tension glaucoma (NTG) (20). Unfortunately, glaucoma can remain asymptomatic until it is quite advanced, hence about 10-50% of patients are unaware that they suffer from this disease (21–25). Although sustained lowering of IOP can slow patients vision loss, it cannot completely stop disease progression (26, 27). Additionally, the administration of topical medications can lead to side effects, such as ocular irritation, blurred vision, or bradycardia, further decreasing compliance of patients (11). These facts emphasize the importance of discovering new treatment strategies for glaucoma patients.

An autoimmune component to its pathology has long been suspected and studies described alterations in antibody titers in glaucoma patients. These changes in antibody titers were found in POAG as well as in NTG patients (28–30). Chen et al. reported that elevated IOP in an animal model can trigger infiltration of autoreactive T-cells into the retina, which cause neurodegeneration by cross-reacting with heat shock protein (HSP)-expressing RGCs. Furthermore, they noted that POAG as well as NTG patients have more HSP27- and HSP60-specific T-cells, indicating that these findings are likely relevant for glaucoma patients (31). Also, altered expressions of proteins of the complement system, as part of the innate immune system, were identified in sera and retinae of glaucoma patients (32, 33) as well as in glaucoma animal models (34–37). Furthermore, an activation of microglia is well known to be involved in the disease pathology (38–40). All this strengthens the hypothesis that immune alterations play an important role in the development of glaucoma.

However, so far, new therapeutic strategies were only investigated in models based on one (patho)mechanism. Therefore, a combination of pathogenic factors in one model would be beneficial and could hopefully mimic this multifactorial disease more precisely. In the βB1-connective tissue growth factor (CTGF) model, the lens-specific CTGF overexpression leads to changes in the extracellular matrix and cytoskeleton in the trabecular meshwork, resulting in an increased IOP. Furthermore, a progressive loss of axons in the CTGF optic nerves was reported after four weeks (41). Additionally, our group noted that an activation of the complement system and a cytokine response takes place before the IOP elevation and accompanied RGC loss at 15 weeks of age (42, 43). To investigate the contribution of the immune system in this disease, we use an autoimmune glaucoma model. Here, a loss of RGCs and optic nerve degeneration is provoked by immunization with antigens, for instance the optic nerve antigen homogenate (ONA) in rats and mice. No IOP alterations are observed in this model (44–48). Furthermore, an increase of the complement system plus an activation of microglia cells prior to cell death were detected (36, 46, 49).

Now, we aimed to implement two pathogenetic factors combined in one glaucoma model. Here, it was evident that the combination of these models led to an increased degeneration, especially in the optic nerves. In addition, a more severe RGCs loss could be noted through this combination.

## 2 Methods

### 2.1 Animals

All procedures concerning animals adhered to the ARVO statement for the use of animals in ophthalmic and vision research. All experiments involving animals were approved by the animal care committee of North Rhine-Westphalia, Germany (file no. 81-02.04.2018.A071).

Mice were kept under environmentally controlled conditions with free access to chow and water. The used transgenic CTGF and wildtype (WT) mice in this study had a CD1 background (42, 43, 50). WT CD1 mice for breeding were obtained from Charles River (Sulzfeld, Germany), while CTGF mice for breeding were kindly provided by Prof. Dr. Fuchshofer (University Regensburg, Germany). Then, all animals for this study were bred and housed at the animal facility at the Ruhr-University Bochum (Bochum, Germany). Potential CTGF mice were screened by isolating genomic DNA from tail biopsies and testing for transgenic sequenced by PCR, using the following primer sequences: 5´-GGAAGTGCCAGCTCATCAGT-3´ and 5´-GTGCGGGACAGAAACCTG-3´. Female and male mice were included in the study.

### 2.2 Immunization

The preparation and immunization of ONA was carried out as previously described (45, 48). Six-week-old WT (=WT+ONA) and CTGF (=CTGF+ONA) mice received an intraperitoneal injection with 1.0 mg/ml ONA. The antigen was mixed with incomplete Freund’s adjuvant (50 µl; Sigma-Aldrich, St. Louis, MO, USA). WT and CTGF animals of the control groups were injected with NaCl in Freund’s adjuvant. Additionally, all mice received 1 µg pertussis toxin (Sigma-Aldrich) intraperitoneally on days zero and two (51).

### 2.3 Measurement of intraocular pressure

IOP of both eyes in all animals was measured before (baseline) and then two, four, and six weeks after immunization using a rebound tonometer (TonoLab, Icare, Vantaa, Finland) as described previously (n=7/group) (46, 52). For this procedure, mice were anesthetized. All measurements were performed by one examiner at the same time of the day. For each analysis, ten measurements per eye were calculated and the average of both eyes was used.

### 2.4 Electroretinogram measurements

Electroretinogram (ERG) measurements were performed six weeks after immunization as previously described (53, 54). Before carrying out the ERG under dim red light, mice were dark adapted overnight. The readings were done using a full-field flash electroretinograph (HMs ERG system, OcuScience, Henderson, NV, USA). After anesthesia with a ketamine (Ratiopharm, Ulm, Germany)/xylazine (Bayer healthcare, Berlin, Germany) cocktail (120/16 mg/kg), eyes were dilated and topically anesthetized. Reference electrodes were placed subcutaneously below the right and left ear and a ground electrode was placed in the base of the tail. Silver thread recording electrodes were placed in the center of the cornea. Scotopic flash ERGs were recorded at 0.1, 0.3, 1, 3, 10, and 25 cd*s/m² (n=6/group). Signals obtained from the corneal surface were then amplified, digitized, and averaged using ERG View 4.380R software (OcuScience). Briefly, the amplitude of the a-wave was measured from the pre-stimulus baseline (0 µV) to the trough of the a-wave. The amplitude of the b-wave was measured from the trough of the a-wave to the peak of the b-wave.

### 2.5 Quantitative real‐time PCR

Both retinae of each animal (n=4 animals/group) were pooled for RNA preparation and cDNA synthesis as previously described (42). For RNA isolation of optic nerves, the ReliaPrep™ RNA Tissue Miniprep System (Promega, Madison, WI, USA) was used (48, 55). The optic nerves of each animal were pooled and incubated in liquid nitrogen prior to isolation and homogenized with a pestle (n=5/group). The designed oligonucleotides for RT-qPCR are shown in **Table 1**. Expression in retina and optic nerve was normalized against *beta-actin (Actb)* and *cyclophilin* (*Ppid*) as well as *Rn18s*, respectively (48). The RT-qPCR was performed using DyNAmo Flash SYBR Green (Fisher Scientific, Schwerte, Germany) on the PikoReal RT-qPCR Cycler (Fisher Scientific) (53, 56). Values were transferred to REST^©^ software (Qiagen, Hilden, Germany) for further analysis.

**Table 1:** Sequences of oligonucleotides.

Gene	Forward (F) and reverse (R) oligonucleotides	GenBank acc. no.	Amplicon size
*Actb*-F *Actb* -R	ctaaggccaaccgtgaaag accagaggcatacagggaca	NM_007393.5	104 bp
*Casp3*-F *Casp3*-R	gaggctgacttcctgtatgctt aaccacgacccgtccttt	NM_001284409.1	77 bp
*Casp7*-F *Casp7*-R	gaccgatgcaaaaccctgtt acctggaaccgtggagtaag	NM_007611.2	180 bp
*Casp8*-F *Casp8*-R	ttgaacaatgagatccccaaa ccatttctacaaaaatttcaagcag	NM_009812.2	70 bp
*Casp9*-F *Casp9*-R	gtacatcgagaccttggatgg tcgcagaaacagcattgg	NM_015733.5	95 bp
*Gfap-F* *Gfap-R*	acagactttctccaacctccag ccttctgacacggatttggt	NM_010277.3	63 bp
*Iba1*-F *Iba1*-R	ggatttgcagggaggaaaa tgggatcatcgaggaattg	D86382.1	92 bp
*Mbp*-F *Mbp*-R	ctttctcagacggcctcaga gactctgagggcctgtcttt	NM_010777.3	239 bp
*Mog*-F *Mog*-R	gagcaagcacctgaataccg caagtgcgatgagagtcagc	NM_010814.2	220 bp
*Olig2-F* *Olig2*-R	ttacagaccgagccaacacc tggccccagggatgatctaa	NM_016967.2	129 bp
*Pou4f1*-F *Pou4f1*-R	ctccctgagcacaagtaccc ctggcgaagaggttgctc	AY706205.1	98 bp
*Ppid*-F *Ppid*-R	ttcttcataaccacaagtcaagacc tccacctccgtaccacatc	M60456.1	95 bp
*Rn18s*-F *Rn18s*-R	gcaattattccccatgaacg gggacttaatcaacgcaagc	NR_003278.3	68 bp
*Tmem119*-F *Tmem119*-R	gtgtctaacaggccccagaa agccacgtggtatcaaggag	NM_146162.3	110 bp
*Vim*-F *Vim*-R	gtggatcagctcaccaacga ctttcatactgctggcgcac	NM_011701.4	353 bp

The listed oligonucleotide pairs were used in quantitative real-time PCR experiments. In the retina *Beta-actin* (*Actb*) and *Cyclophilin* (*Ppid*) and in the optic nerve *Rn18s* served as housekeeping genes. The predicted amplicon sizes are given. F, forward; R, reverse; acc. no., accession number; bp, base pair.

### 2.6 Tissue preparation for (immuno-)histology

Six weeks after immunization, optic nerves and eyes were enucleated and fixed in 4% paraformaldehyde for 2 h (optic nerves) or 1 h (eyes). Thereafter, the tissues underwent a 30% sucrose treatment and got embedded in a Neg-50 compound (Tissue-Tek; Fisher Scientific). Longitudinal sections of the optic nerve (4 µm) and cross-sections of the retina (10 µm) were cut with a cryostat (Fisher Scientific) for further staining (57).

### 2.7 Histopathological stainings

To evaluate the extent of cellular infiltration, longitudinal cryo-sections of optic nerves (n=8/group) were stained with hematoxylin & eosin (H&E; Merck Millipore, Burlington, MA, USA). The degree of demyelination was examined *via* luxol fast blue (LFB; RAL Diagnostics, Martillac Cedex, France) (58). After the stainings, ethanol was used for dehydration of the sections, followed by incubation in xylene (Merck Millipore) and coating with Eukitt (VWR, Langenfeld, Germany).

Three images of each optic nerve section (anterior, medial, and posterior) were taken with an Axio Imager M1 microscope (Zeiss, Jena, Germany) at a 400x magnification (six sections per animal).

The masked pictures were evaluated by two independent examiners regarding the extent of inflammatory cell infiltration using an established score (59, 60): 0=no infiltration, 1=mild cellular infiltration, 2=moderate infiltration, 3=severe infiltration, and 4=massive infiltration with formation of cellular conglomerates (**Supplementary Figure 1A**). Regarding the degree of demyelination, LFB-stained sections were assessed as previously described (60–62): 0=no demyelination, 0.5=mild demyelination, 1=moderate demyelination, 1.5=advanced demyelination, and 2=severe demyelination up to complete loss of structural integrity (**Supplementary Figure 1B**).

Retinal cross-sections (n=5/group) were stained with Nissl to evaluate the number of neurons in the ganglion cell layer (GCL) (57, 63). Briefly, after de- and rehydration in 70 to 100% ethanol, cross-sections were stained with the 1% cresyl violet (Merck Millipore). Then, all slides were again dehydrated in ethanol followed by incubation in xylene and coated with Eukitt. Two photos of the peripheral and two of the central part of each retinal cross-section were captured at a 400x magnification (Axio Imager M1).

### 2.8 Immunohistology

In order to identify different cell types, specific immunofluorescence antibodies were applied (**Table 2**) (36). Briefly, optic nerve longitudinal and retina cross-sections (both: n=8/group) were blocked with a solution containing 10-20% donkey, 2-3% bovine serum albumin, and/or goat serum, and 0.1% Triton-X in PBS. Sections were incubated with primary antibodies at room temperature overnight. Incubation using corresponding secondary antibodies was performed for 1 h on the next day. Apoptotic cells in the optic nerves were visualized by using a TdT-mediated dUTP-biotin nick end labeling (TUNEL) assay (n=5/group) and labeling was carried out according to the manufacturer’s instructions (*In Situ* Cell Death Detection Kit TMR red, Roche, Sigma‐Aldrich). Nuclear staining with 4´,6 diamidino-2-phenylindole (DAPI, Serva Electrophoresis, Heidelberg, Germany) was included to facilitate the orientation on the slides. Negative controls were performed for each stain by using secondary antibodies only.

**Table 2:** Primary and secondary antibodies used for immunohistology on optic nerve and retina sections.

Primary antibodies				Secondary antibodies			
Antibody	Company	Dilution	Tissue	Antibody	Company	Dilution	Tissue
CC1	Abcam	1:200	Optic nerve	Donkey anti-mouse Alexa Fluor 488	Invitrogen	1:500	Optic nerve
Cleaved caspase 3	Sigma-Aldrich	1:100	Optic nerve	Donkey anti-rabbit Alexa Fluor 555	Invitrogen	1:400	Optic nerve
GFAP	Merck Millipore	1:400	Optic nerve	Donkey anti-chicken Cy3	Invitrogen	1:500	Optic nerve
Iba1	Synaptic System	1:500	Optic nerve	Donkey anti-chicken Cy3	Sigma-Aldrich	1:400	Optic nerve
			Retina				Retina
Olig2	Millipore	1:500	Optic nerve	Donkey anti-rabbit Alexa Fluor 555	Invitrogen	1:500	Optic nerve
RBPMS	Millipore	1:500	Retina	Donkey anti-rabbit Alexa Fluor 555	Invitrogen	1:500	Retina
S100B	Novus Biological	1:100	Optic nerve	Goat anti-rabbit Alexa Fluor 488	Invitrogen	1:500	Optic nerve
Tmem119	Abcam	1:200	Optic nerve	Donkey anti-rabbit Alexa Fluor 488	Jackson ImmunoResearch	1:500	Optic nerve
			Retina				Retina
Vimentin	Sigma-Aldrich	1:500	Retina	Goat anti-mouse Alexa Fluor 488	Invitrogen	1:500	Retina

### 2.9 Histological examination

The photographs were taken using a fluorescence microscope (Axio Imager M1 or M2). Two photos of the peripheral and two of the central part of each retinal cross-section at a distance of 300 and 3100 µm dorsal and ventral to the optic nerve were captured (in total 24 images/animal/). Three images of each optic nerve section (anterior, medial, and posterior) were obtained (in total 18 images/animal). The images were transferred to Corel Paint Shop Pro (V13, Corel Corporation, Ottawa, Canada) and equal excerpts were cut out. Afterwards, Nissl^+^, RBPMS^+^, Iba1^+^, Tmem-119^+^ and Iba1^+^, cleaved caspase 3^+^, and TUNEL^+^ cells were counted using ImageJ software (NIH, Bethesda, MD, USA) (42). RBPMS^+^ cells were counted in total as well as separately in the central and peripheral part of each cross-section. In the optic nerve, Iba1^+^ as well as Tmem119^+^ and Iba1^+^ co-localized cells were counted. In the retina, Iba1^+^ as well as Iba1^+^ and Tmem119^+^ co-localized cells were counted in the GCL only and in the GCL, inner plexiform layer (IPL), inner nuclear layer (INL), and outer plexiform layer (OPL) together (GCL-OPL). All immunohistological cell markers were counted only if they were co-localized with DAPI.

Vimentin^+^ staining area was evaluated using an ImageJ software macro. Briefly, images were transformed into grayscale. To minimize interference with background labeling, a defined rolling ball radius of 50 pixels was subtracted. The percentage (%) of the labeled area was then measured between defined thresholds, which were obtained when the grayscale and the original picture corresponded the most (lower threshold: 6.02; upper threshold: 162.12) (57, 64).

Glial fibrillary acid protein (GFAP) and S100B staining in the optic nerves were evaluated using a scoring system, which is modified from an established score for neurofilaments (49, 65, 66): 0=astrocyte structure intact, 0.5=mild astrocyte structure loss, 1=moderate astrocyte structure loss, 1.5=advanced structure loss, and 2=severe up to complete loss of astrocyte structural integrity (**Supplementary Figures 1C, D**).

### 2.10 Statistics

Regarding RT-qPCR, the relative expression values are presented as median ± quartile ± minimum/maximum and were assessed *via* Pair Wise Fixed Reallocation Randomisation Test using REST^©^ software (Qiagen) (42, 47, 67). For IOP, ERG, histology, and immunofluorescence, groups were compared by ANOVA followed by Tukey Honest *post-hoc* test (Statistica Software; Version 13, Dell, Tulsa, OK, USA) and are presented as mean ± SEM. H&E, LFB, GFAP, and S100B optic nerves scores comprised Kruskal-Wallis test followed by Dunn’s test using Graph Pad Prism 9.4.1 (San Diego, CA, USA) and are presented as median ± interquartile range (IQR) ± range (56). P-values below 0.05 were considered statistically significant, with *p<0.05, **p<0.01, and ***p<0.001 when compared to WT; ^#^p<0.05, ^##^p<0.01, and ^###^p<0.001 when compared to WT+ONA, ^¥^p<0.05, ^¥¥^p<0.01, and ^¥¥¥^p<0.001 when compared to CTGF, and ^$^p<0.05 when compared to CTGF+ONA.

## 3 Results

### 3.1 Mild elevated intraocular pressure in CTGF and CTGF+ONA mice

It is known that CTGF mice develop a significantly increased IOP at the age of 15 weeks (42). After ONA immunization, in the autoimmune glaucoma model, the IOP stays within the normal range (48, 68).

**Figure 1:**
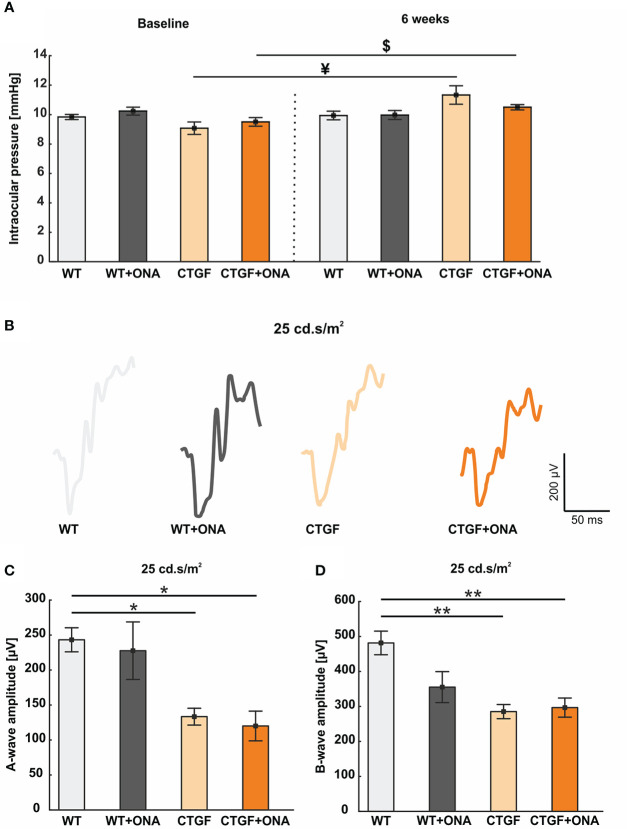
Mild increase of IOP and loss of retinal function. **(A)** The IOP measurements before (baseline) and six weeks after immunization are displayed. At baseline, the IOP was comparable in all groups. Also, no changes could be observed six weeks after immunization between the groups. However, the IOP of the CTGF group was significantly higher at six weeks compared to CTGF animals at baseline. Additionally, a significant higher IOP could be noted in CTGF+ONA mice at six weeks compared to baseline. **(B)** Exemplary ERG amplitudes at 25 cd*m/s^2^ flash luminance six weeks after immunization. **(C)** The a-wave amplitudes at 25 cd*m/s^2^ were significantly lower in CTGF and CTGF+ONA mice compared to controls. **(D)** Also, a loss of the electrical output of the b-wave at 25 cd.s/m^2^ was noted in CTGF and CTGF+ONA mice compared to WT. Values are mean ± SEM. *p<0.05 and **p<0.01 *vs*. WT; ^¥^p<0.05 *vs*. CTGF; ^$^p<0.05 *vs*. CTGF+ONA.

In the current study, the IOP at the baseline was not different between WT, WT+ONA, CTGF, and CTGF+ONA mice (all: p>0.05; **Table 3**). Further, the IOP was not altered within the groups at two, four, and six weeks (all: p>0.05; **Table 3**; **Supplementary Figure 2A**). Six weeks after immunization, at the age of twelve weeks, the IOP of the CTGF mice was significantly higher when compared to the IOP values of this group at baseline (p=0.013; **Figure 1A**). The mean IOP in the WT+ONA group was not different between all points in time (all: p>0.05). The IOP of the CTGF+ONA group was significantly elevated when compared to baseline values (p=0.027; **Figure 1A**).

**Table 3:** Intraocular pressure values throughout the study.

Week	Mean ± SEM [mmHg]				P-value					
	WT	WT+ONA	CTGF	CTGF+ONA	WT *vs.* WT+ONA	WT *vs.* CTGF	WT *vs.* CTGF+ONA	WT+ONA *vs.* CTGF	WT+ONA *vs.* CTGF+ONA	CTGF *vs.* CTGF+ONA
**0**	9.84 ± 0.17	10.24 ± 0.27	9.08 ± 0.42	9.51 ± 0.29	0.796	0.305	0.861	0.056	0.345	0.750
**2**	10.30 ± 0.09	10.47 ± 0.26	9.70 ± 0.39	9.72 ± 0.23	0.968	0.401	0.432	0.199	0.219	1.000
**4**	10.45 ± 0.29	10.61 ± 0.10	9.63 ± 0.42	9.51 ± 0.24	0.977	0.202	0.121	0.095	0.053	0.992
**6**	9.94 ± 0.29	9.97 ± 0.16	11.33 ± 0.63	10.51 ± 0.16	1.000	0.078	0.720	0.087	0.753	0.457

P-value						
**Group**	**0 *vs.* 2 weeks**	**0 *vs*. 4 weeks**	**0 *vs*. 6 weeks**	**2 *vs*. 4 weeks**	**2 *vs.* 6 weeks**	**4 *vs.* 6 weeks**
**WT**	0.501	0.232	0.991	0.966	0.681	0.406
**WT+ONA**	0.904	0.700	0.872	0.976	0.500	0.277
**CTFG**	0.791	0.844	**0.013**	1.000	0.098	0.080
**CTGF+ONA**	0.916	1.000	**0.027**	0.923	0.106	**0.028**

Significant p-values are marked in bold. Values are mean ± SEM.

Taken together, CTGF and CTGF+ONA animals developed an increased IOP over time.

### 3.2 Slight loss of retinal function

ERGs were performed six weeks after immunization. The a-wave amplitude reflects the response of the photoreceptors. At the light intensities from 0.1 to 10 cd*m/s^2^, no changes could be detected within the groups (all: p>0.05; **Table 4**; **Supplementary Figure 2B**). At 25 cd*m/s^2^, the a-wave amplitude was lower in CTGF (p=0.029) and CTGF+ONA eyes (p=0.013) compared to WT. No differences were noted between CTGF and WT+ONA mice (p=0.071), while the a-wave amplitude was lower in CTGF+ONA retinae compared to WT+ONA animals (p=0.034; **Table 4**; **Figures 1B, C**).

**Table 4:** Summary of ERG results.

A-wave amplitude										
cd*m/s^2^	Mean ± SEM [µV]				P-value					
	WT	WT+ONA	CTGF	CTGF+ONA	WT *vs.* WT+ONA	WT *vs.* CTGF	WT *vs.* CTGF+ONA	WT+ONA *vs.* CTGF	WT+ONA *vs.* CTGF+ONA	CTGF *vs.* CTGF+ONA
**0.1**	101.83 ± 22.03	74.78 ± 16.97	54.29 ± 11.69	44.17 ± 5.20	0.604	0.158	0.066	0.781	0.506	0.965
**0.3**	123.83 ± 39.64	96.59 ± 16.11	78.41 ± 12.21	69.89 ± 14.45	0.828	0.512	0.370	0.946	0.850	0.994
**1**	172.62 ± 51.70	135.49 ± 19.25	100.88 ± 11.81	85.87 ± 9.01	0.795	0.314	0.172	0.827	0.615	0.982
**3**	176.62 ± 33.73	175.11 ± 33.83	97.73 ± 14.18	93.88 ± 18.81	1.000	0.186	0.156	0.199	0.167	1.000
**10**	233.89 ± 19.00	194.18 ± 41.00	134.93 ± 24.02	133.13 ± 26.04	0.883	0.160	0.150	0.479	0.454	1.000
**25**	243.21 ± 17.20	227.64 ± 41.13	133.39 ± 12.04	120.08 ± 21.24	0.972	**0.029**	**0.013**	0.071	**0.034**	0.982
B-wave amplitude										
cd*m/s^2^	Mean ± SEM [µV]				P-value					
	WT	WT+ONA	CTGF	CTGF+ONA	WT *vs.* WT+ONA	WT *vs.* CTGF	WT *vs.* CTGF+ONA	WT+ONA *vs.* CTGF	WT+ONA *vs.* CTGF+ONA	CTGF *vs.* CTGF+ONA
**0.1**	300.62 ± 74.40	253.40 ± 15.69	185.78 ± 22.21	207.00 ± 19.05	0.845	0.224	0.388	0.650	0.851	0.983
**0.3**	377.29 ± 83.70	351.89 ± 29.82	228.35 ± 19.12	247.73 ± 19.99	0.980	0.141	0.233	0.269	0.410	0.991
**1**	428.67 ± 101.11	347.78 ± 32.49	248.38 ± 17.74	249.14 ± 32.45	0.745	0.141	0.144	0.604	0.610	1.000
**3**	367.48 ± 66.62	334.68 ± 20.33	269.18 ± 21.81	256.18 ± 25.76	0.931	0.304	0.209	0.636	0.494	0.995
**10**	437.55 ± 42.04	371.88 ± 44.56	298.45 ± 36.48	309.28 ± 32.84	0.644	0.090	0.129	0.560	0.677	0.997
**25**	581.51 ± 33.77	355.25 ± 44.21	285.26 ± 20.26	296.72 ± 27.35	0.057	**0.002**	**0.004**	0.446	0.592	0.995

For all light intensities, the mean a- and b-wave amplitudes of all groups and the respective p-values are displayed. Significant p-values are marked in bold. Values are mean ± SEM.

The b-wave largely reflects ON bipolar cell responses. Similar to the results of the a-wave amplitude, no difference could be detected in the b-wave amplitudes at the light intensities from 0.1 to 10 cd*m/s^2^ (all: p>0.05; **Table 4**; **Supplementary Figure 2C**). At 25 cd*m/s^2^, the b-wave amplitude was significantly lower in CTGF (p=0.002) as well as in CTGF+ONA mice (p=0.004) when compared to WT. No alterations were noted when comparing WT+ONA, CTGF, and CTGF+ONA eyes with each other (all: p>0.05, **Table 4**; **Figures 1B, D**).

To sum up, the electrical output was diminished at the highest amplitude in CTGF and CTGF+ONA mice.

### 3.3 Increased cell infiltration and demyelination in CTGF+ONA group

**Figure 2:**
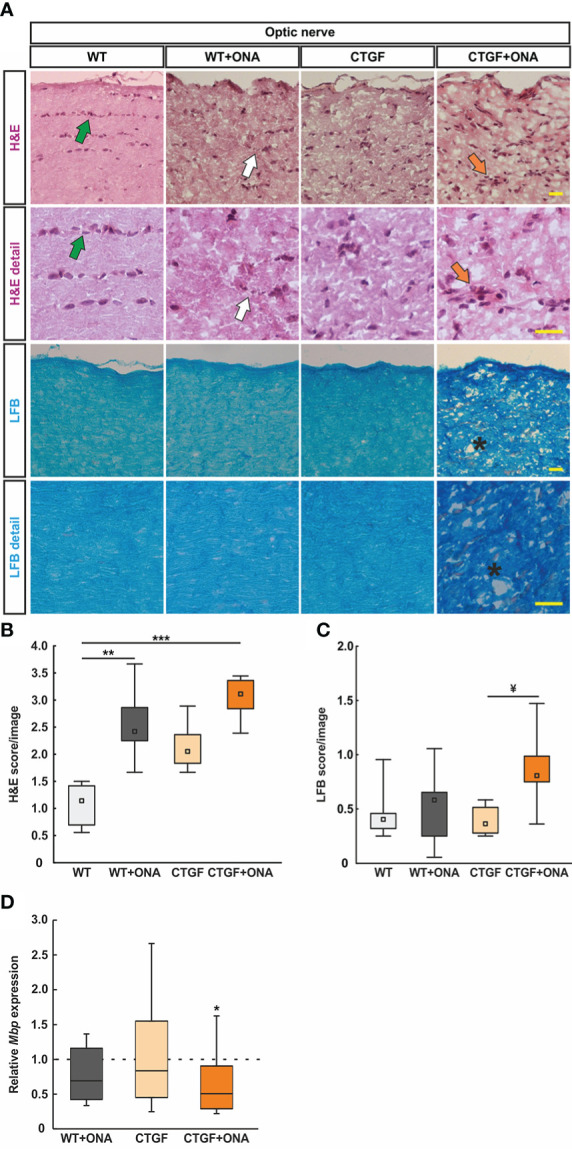
Enhanced optic nerve degeneration. **(A)** Longitudinal optic nerve sections were stained with H&E (cellular inflammation) and LFB (demyelination) and are shown as overview and in detail. In the H&E staining, healthy optic nerves show a linear formation of the nuclei (green arrows), which is disrupted in glaucomatous optic nerves (white arrows) and might comprise cellular conglomerates (orange arrows). LFB stained myelin sheats in healthy optic nerves resembles combed bundles in a parallel arrangement, which is interrupted in CTGF+ONA optic nerves (stars). **(B)** The H&E score revealed a higher degree of cellular inflammation in WT+ONA and CTGF+ONA animals compared to WT. **(C)** The LFB score showed a disruption of myelin in CTGF+ONA optic nerves compared to CTGF animals. **(D)** The mRNA expression levels of *Mbp* were significantly downregulated in CTGF+ONA optic nerves compared to controls. Values are median +/- interquartile range +/- range for histology and median ± quartile ± minimum/maximum for RT-qPCR. The dotted line in **(D)** represents the relative expression of the WT group. *p<0.05, **p<0.01 and ***p<0.001 *vs*. WT; and ^¥^p<0.05 *vs*. CTGF. Scale bars: 20 µm.

A possible cell infiltration was determined by H&E scoring of longitudinal optic nerve sections at six weeks (**Figure 2A**). The WT+ONA group showed significantly more cell infiltrations with a median score of 2.42 (IQR 2.24-3.67) than WT animals with a score of 1.14 (IQR 0.65-1.43; p=0.006). CTGF optic nerves did not reveal a significantly higher score (2.06, IQR 1.75-2.43; p=0.150). However, the CTGF+ONA optic nerves revealed a significant higher score compared to WT (3.11, IQR 2.74-3.68; p<0.001). No difference was noted between CTGF and WT+ONA animals (p=1.000). The score of the CTGF+ONA group was not altered compared to WT+ONA mice (p=0.993) but showed a mild trend towards a higher score in comparison to CTGF optic nerves (p=0.084; **Figure 2B**).

Demyelination processes were examined *via* LFB staining and subsequent scoring (**Figure 2A**). WT+ONA animals (0.58, IQR 0.24-0.66) showed similar scores as WT ones (0.40, IQR 0.30-0.47; p=1.000). A comparable LFB score was revealed in CTGF optic nerves (0.36, IQR 0.26-0.52) compared to WT and WT+ONA animals (both: p=1.000). A trend towards a higher demyelination score was observable in CTGF+ONA optic nerves with a score of 0.81 (IQR 0.72-0.99) compared to WT mice (p=0.054). No changes were noted when comparing CTGF+ONA to WT+ONA animals (p=0.225), while a significantly higher score was revealed compared to CTGF optic nerves (p=0.020; **Figure 2C**).

In addition, mRNA levels of *Mbp* (myelin binding protein) were evaluated *via* RT-qPCR. The *Mbp* mRNA levels in WT+ONA (0.69-fold expression; p=0.136) and CTGF optic nerves (0.84-fold expression; p=0.584) did not differ from WT nerves. In accordance with the LFB staining, a significant downregulation of *Mbp* mRNA levels could be observed in CTGF+ONA optic nerves (0.51-fold expression; p=0.044; **Figure 2D**).

In summary, the combination of two risk factors led to more inflammatory cells and a more pronounced damage of myelin.

### 3.4 Loss of oligodendrocytes predominantly in WT+ONA optic nerves

**Figure 3:**
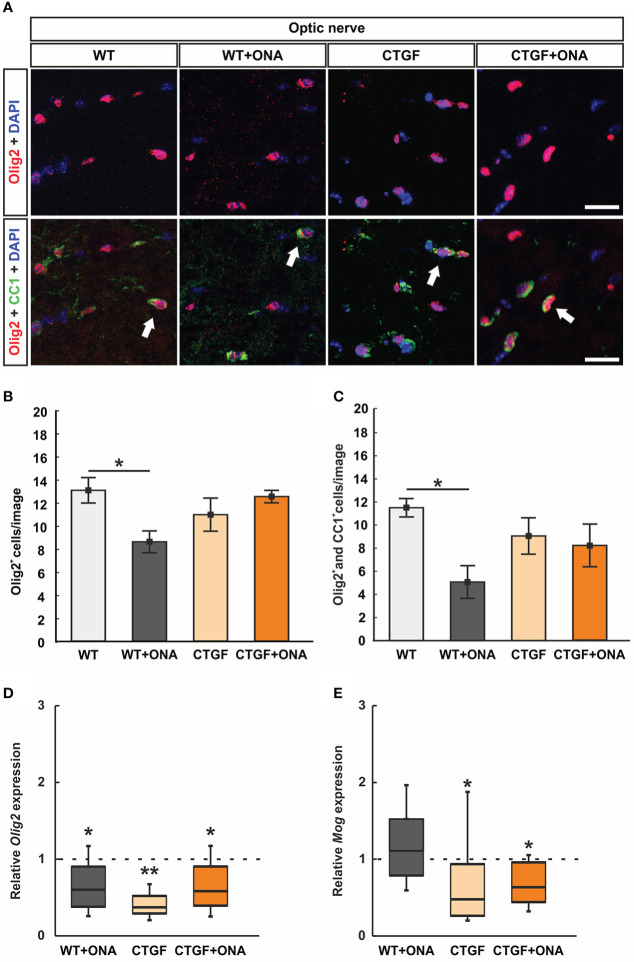
Loss of oligodendrocytes in WT+ONA mice. **(A)** Optic nerve sections were labelled with antibodies against anti-Olig2 (red) and anti-CC1 (green). Single Olig2^+^ cells were identified as immature oligodendrocytes, while a co-localization of Olig2 and CC1 double positive cells identified mature oligodendrocytes (arrows). **(B)** The number of single Olig2^+^ cells was significantly decreased in WT+ONA optic nerves compared to WT mice, while no alterations were noted in the CTGF and CTGF+ONA groups. **(C)** The number of Olig2^+^ and CC1^+^ double positive cells was significantly diminished in the WT+ONA animals compared to controls. No changes were observed within the other groups. **(D)** The mRNA expression levels of *Olig2* were significantly decreased in WT+ONA, CTGF, and CTGF+ONA optic nerves compared to WT. **(E)** No alterations were noted in the mRNA expression levels of *Mog* in the WT+ONA group. In contrast, a significant downregulation could be observed in CTGF and CTGF+ONA optic nerves compared to WT. Values are mean ± SEM for immunohistology and median ± quartile ± minimum/maximum for RT-qPCR. The dotted lines in **(D, E)** represent the relative expression of the WT group. *p<0.05 and **p<0.01 *vs*. WT. Scale bars: 20 µm.

Since oligodendrocytes appear in two different populations, immature and mature, immunohistological staining was done with antibodies against Olig2, which is expressed by oligodendrocytes of all stages (69), and CC1, expressed by mature oligodendrocytes (70) (**Figure 3A**). Hence, single Olig2^+^ cells were identified as immature and co-localization identified double positive cells as mature oligodendrocytes (48).

The number of single Olig2^+^ cells was significantly diminished in WT+ONA optic nerves (8.59 ± 0.95 cells/image) compared to WT ones (13.05 ± 1.10 cells/image; p=0.030). The number of Olig2^+^ cells was comparable in CTGF (10.95 ± 1.43 cells/image; p=0.506) or CTGF+ONA mice (12.511 ± 0.54 cells/image; p=0.983) when compared to WT optic nerves. Also, no difference was observed between CTGF and WT+ONA optic nerves (p=0.409). In addition, no changes were noted when comparing CTGF+ONA nerve sections to WT+ONA (p=0.066) or CTGF mice (p=0.724; **Figure 3B**).

Significantly fewer Olig2^+^ and CC1^+^ mature oligodendrocytes were noted in WT+ONA optic nerves (5.07 ± 1.42 cells/image; p=0.023) in comparison to WT (11.51 ± 0.80 cells/image). In contrast, no changes in the number of double positive cells were observed in CTGF (9.06 ± 1.57 cells/image; p=0.643) and CTGF+ONA mice (8.19 ± 1.85 cells/image; p=0.396) compared to WT ones. Also, no differences were noted amongst CTGF and WT+ONA optic nerves (p=0.243). The comparison between CTGF+ONA and WT+ONA (p=0.447) or CTGF optic nerves (p=0.975) revealed no alterations in the Olig2^+^ and CC1^+^ cell number (**Figure 3C**).

Furthermore, the mRNA levels of *Olig2* (oligodendrocyte transcription factor 2) were analyzed with RT-qPCR. In accordance with the immunohistological results, a significant downregulation of *Olig2* mRNA levels was noted in WT+ONA optic nerves (0.60-fold expression; p=0.038). Further, the mRNA levels of *Olig2* were downregulated in CTGF (0.37-fold expression; p=0.004) and CTGF+ONA animals (0.58-fold expression; p=0.039) compared to WT (**Figure 3D**).

Additionally, *Mog* (myelin oligodendrocyte glycoprotein) mRNA levels were examined. The mRNA expression of *Mog* was not different in WT+ONA optic nerves compared to WT (1.11-fold expression; p=0.556). In contrast, a significant downregulation of *Mog* mRNA levels was observed in CTGF (0.48-fold expression; p=0.040) as well as in CTGF+ONA mice (0.64-fold expression; p=0.038; **Figure 3E**).

Overall, the number of oligodendrocytes was diminished only in WT+ONA optic nerves, while mRNA expression levels were altered in all glaucoma groups.

### 3.5 Additive macrogliosis in CTGF+ONA optic nerves

**Figure 4:**
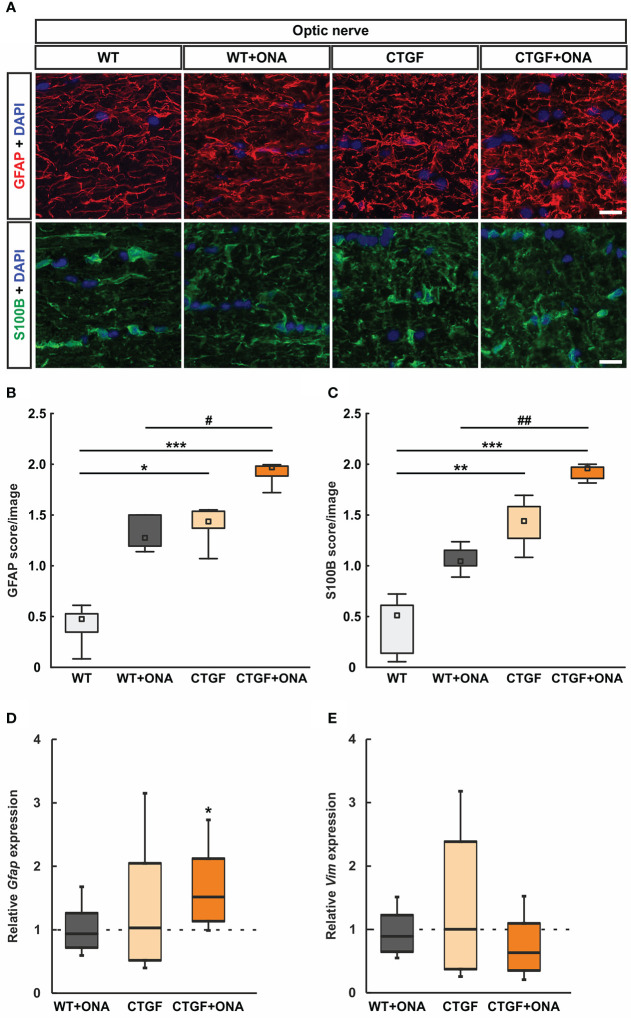
Increased astrogliosis in CTGF+ONA optic nerves. **(A)** Longitudinal optic nerve sections were stained with antibodies against anti-GFAP (red) and anti-S100B (green) to label astrocytes. DAPI was used to counterstain cell nuclei (blue). **(B)** The GFAP score was not altered in WT+ONA optic nerves compared to WT. A higher score was revealed in CTGF and CTGF+ONA optic nerves compared WT. The GFAP score in CTGF+ONA animals was also significantly higher than in WT+ONA mice. **(C)** In WT+ONA animals, the S100B score was not different when compared to WT optic nerves. A significantly higher S100B score was revealed in CTGF and CTGF+ONA optic nerves compared to WT. The S100B score of CTGF+ONA animals was significantly higher than in WT+ONA optic nerves. **(D)** The mRNA expression levels of *Gfap* were not altered in WT+ONA and CTGF mice. In contrast, a significant upregulation was noted in CTGF+ONA optic nerves. **(E)** The mRNA expression levels of *Vim* were similar in all groups. Values are median +/- interquartile range +/- range for histology and median ± quartile ± minimum/maximum for RT-qPCR. The dotted lines in **(D, E)** represent the relative expression of the WT group. *p<0.05, **p<0.01, and ***p<0.001 *vs*. WT; ^#^p<0.05 and ^##^p<0.01 *vs*. WT+ONA. Scale bars: 20 µm.

Optic nerves astrocytes were labelled with an antibody against GFAP (**Figure 4A**). WT+ONA optic nerves, with a median score of 1.27 (IQR 1.19-1.5), showed no differences in the GFAP staining compared to WT animals (0.47, IQR 0.30-0.54; p=0.145). In contrast, CTGF (1.44, IQR 1.35-1.55; p=0.025) as well as CTGF+ONA animals (1.97, IQR 1.86-1.99; p<0.001) revealed a strong GFAP macrogliosis in comparison to WT tissue. No alterations in GFAP scores could be noted between CTGF and WT+ONA sections (p=1.000). The GFAP score of the CTGF+ONA group was significantly increased compared to WT+ONA optic nerves (p=0.025), but not to CTGF mice (p=0.145; **Figure 4B**).

In addition, optic nerves were stained against S100B, as a further astrocyte marker (**Figure 4A**). The mean scores of the WT+ONA with 1.04 (IQR 1.00-1.16) was not altered compared to the WT score of 0.51 (IQR 0.11-0.63; p=0.419). Contrary, the CTGF group (1.44, IQR 1.25-1.58) revealed a significantly higher S100B score then the WT group (p=0.006). Furthermore, a significantly stronger S100B macrogliosis score was revealed in CTGF+ONA optic nerves (1.92, IQR 1.85-1.97) when compared to WT (p<0.001) and WT+ONA (p=0.006) but not compared to CTGF animals (p=0.419; **Figure 4C**).

The mRNA levels of *Gfap* were evaluated through RT-qPCR analysis. No differences in the mRNA expression levels could be noted in WT+ONA (0.94-fold expression; p=0.686) and CTGF optic nerves (1.03-fold expression; p=0.926) compared to WT ones. In contrast, a significant upregulation in *Gfap* mRNA levels was noted in CTGF+ONA optic nerves (1.52-fold expression; p=0.015; **Figure 4D**).

Further, the mRNA expression levels of *Vim* (vimentin) were examined. Here, the mRNA expression levels were not altered in WT+ONA (0.89-fold expression; p=0.446), CTGF (1.02-fold expression; p=0.947) as well as CTGF+ONA optic nerves (0.63-fold expression; p=0.129) when compared to WT (**Figure 4E**).

Summarized, a more pronounced macrogliosis could be revealed in CTGF+ONA optic nerves compared to WT+ONA and CTGF mice alone.

### 3.6 No microglia activation in optic nerves

**Figure 5:**
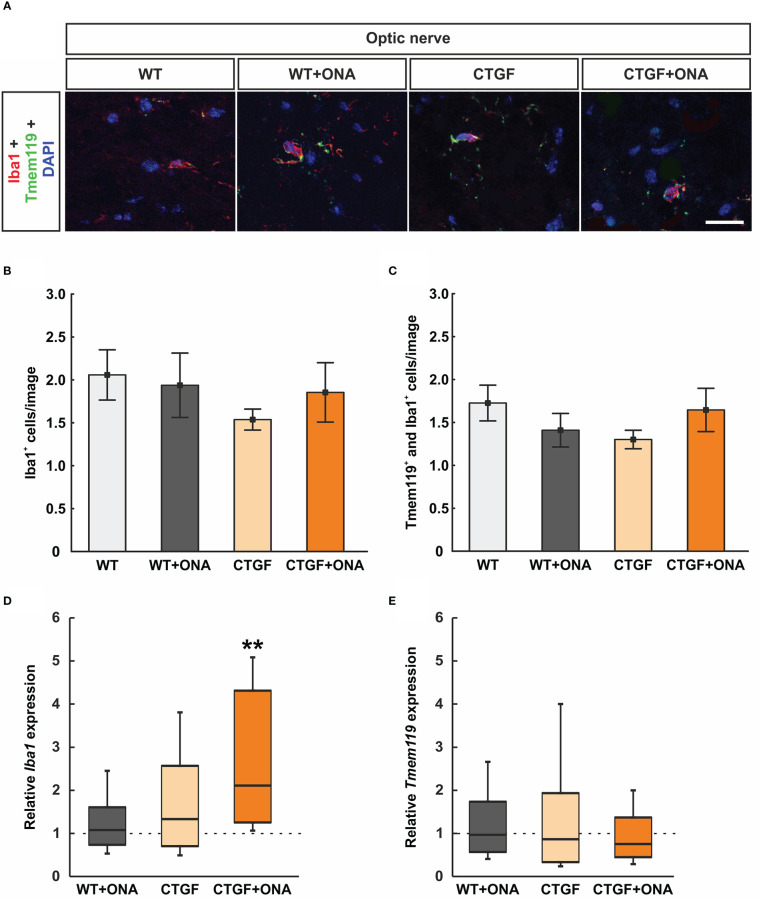
No microglia activation in CTGF+ONA optic nerves **(A)** Microglia/macrophages were labelled with anti-Iba1 (red) and microglia with double staining with anti-Tmem119 (green). DAPI counterstained cell nuclei (blue). **(B)** The number of Iba1^+^ cells was not different within all groups. **(C)** Also, no changes were observed in the number of Tmem119^+^ and Iba1^+^ double positive cells within all groups. **(D)** The mRNA expression levels of *Iba1* were not altered in WT+ONA and CTGF optic nerves, while a significant upregulation was noted in the CTGF+ONA group. **(E)** *Tmem119* mRNA expression levels were equivalent in all groups. Values are mean ± SEM for immunohistology and median ± quartile ± minimum/maximum for RT-qPCR. The dotted lines in **(D, E)** represent the relative expression of the WT group. **p<0.001 *vs*. WT. Scale bars: 20 µm.

Microglia/macrophages were labelled with anti-Iba1 and microglia using anti-Iba1 in combination with an antibody against Tmem119 (**Figure 5A**). The number of Iba1^+^ cells was comparable in WT+ONA (1.94 ± 0.38 cells/image) and WT optic nerves (2.06 ± 0.29 cells/image; p=0.992). Further, no changes were noted in CTGF (1.54 ± 0.12 cells/image; p=0.617) and CTGF+ONA mice (1.85 ± 0.35 cells/image; p=0.963) in comparison to WT. No alterations could be revealed in the number of Iba1^+^ cells in CTGF optic nerves compared to WT+ONA ones (p=0.783) as well as in CTGF+ONA animals compared to WT+ONA (p=0.997) or CTGF optic nerves (p=0.878; **Figure 5B**).

Tmem119^+^ and Iba1^+^ cells (microglia) were also evaluated. Here, the number of double positive cells remained similar within the groups (WT+ONA: 1.41 ± 0.20 cells/image; p=0.674; CTGF: 1.30 cells/image; p=0.441; CTGF+ONA: 1.65 ± 0.25 cells/image; p=0.992) when compared to WT nerves (1.73 ± 0.21 cells/image). Also, no difference was noted when comparing CTGF and WT+ONA optic nerves (p=0.980). Further, no alterations were observed in CTGF+ONA optic nerves regarding Tmem119^+^ and Iba1^+^ cells when compared to WT+ONA (p=0.833) or CTGF ones (p=0.613; **Figure 5C**).

The mRNA expression levels of *Iba1* were evaluated *via* RT-qPCR. No differences were noted in WT+ONA (1.08-fold expression; p=0.732) and CTGF optic nerves (1.33-fold expression; p=0.362). A significant upregulation of *Iba1* mRNA levels was observed in CTGF+ONA animals (2.11-fold expression; p=0.001; **Figure 5D**).

In addition, the mRNA levels of *Tmem119* were examined. Here, consistent with the immunohistological analysis, the expression levels of *Tmem119* mRNA did not differ from control levels in WT+ONA (0.97-fold expression; p=0.934), CTGF (0.87-fold expression; p=0.749), and CTGF+ONA optic nerves (0.76-fold expression; p=0.382; **Figure 5E**).

In summary, no increased microgliosis was notable in all glaucoma optic nerves.

### 3.7 Increased apoptosis in optic nerves

**Figure 6:**
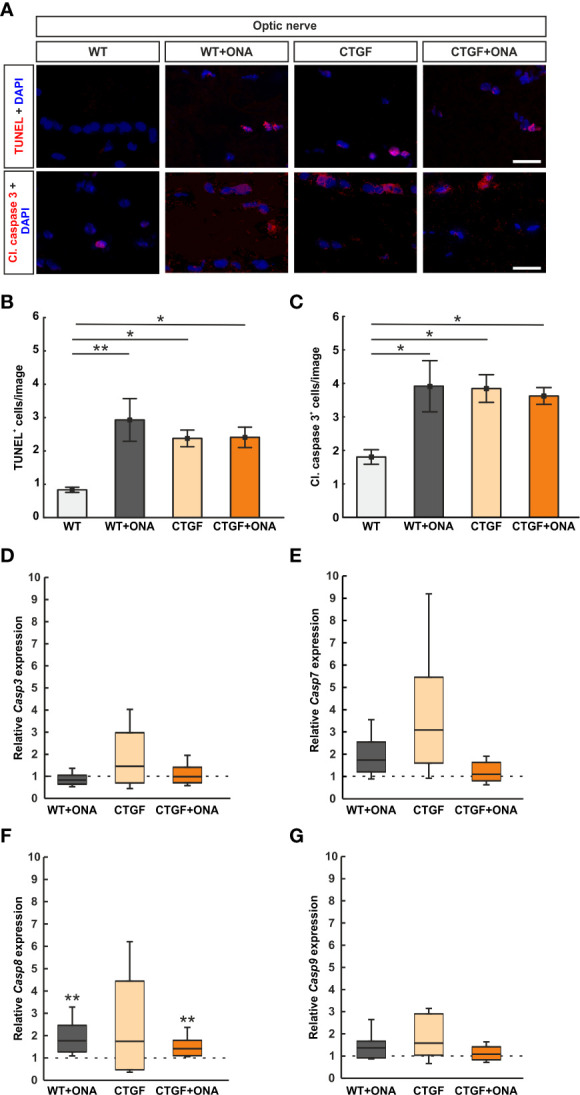
More apoptotic cells in glaucoma optic nerves. **(A)** Apoptotic cells were labelled with TUNEL (red) and an antibody against anti-cleaved caspase 3 (red) on optic nerve sections, while cell nuclei were stained with DAPI (blue). **(B)** The number of TUNEL^+^ cells significantly increased in WT+ONA, CTGF, and CTGF+ONA optic nerves in comparison to WT mice. **(C)** The number of cleaved caspase 3^+^ cells was elevated in WT+ONA, CTGF, and CTGF+ONA mice compared to WT. **(D)** The mRNA expression levels of *Casp3* were not altered. **(E)** *Casp7* mRNA expression levels did not differ between groups. **(F)** The mRNA expression levels of *Casp8* were significantly upregulated in WT+ONA and CTGF+ONA animals. In CTGF optic nerves, the *Casp8* mRNA levels were not altered. **(G)** *Casp9* mRNA expression levels were equivalent in all groups. Values are mean ± SEM for immunohistology and median ± quartile ± minimum/maximum for RT-qPCR. The dotted lines in **(C–F)** represent the relative expression of the WT group. *p<0.05 and **p<0.01 *vs*. WT. Scale bars: 20 µm.

To identify the number of apoptotic cells, optic nerves were stained with TUNEL (**Figure 6A**). The number of TUNEL^+^ cells was significantly increased in WT+ONA mice (2.93 ± 0.64 cells/image) when compared to WT controls (0.83 ± 0.08 cells/image; p=0.006). Also, significantly more TUNEL^+^ cells were observed in CTGF (2.38 ± 0.25 cells/image; p=0.047) as well as CTGF+ONA optic nerves (2.41 ± 0.31 cells/image; p=0.043) in comparison to WT mice. No alterations were revealed when comparing TUNEL^+^ apoptotic cells in CTGF+ONA animals to WT+ONA (p=0.765) as well as CTGF mice (p=1.000; **Figure 6B**).

Optic nerve sections were further labelled against cleaved caspase 3 to detect possible apoptosis (**Figure 6A**). The number of cleaved caspase 3^+^ cells was significantly increased in WT+ONA optic nerves (3.91 ± 0.76 cells/image) compared to WT (1.80 ± 0.22 cells/image; p=0.016). Moreover, significantly more apoptotic cells were noted in CTGF (3.85 ± 0.41 cells/image; p=0.026) and CTGF+ONA mice (3.62 ± 0.25 cells/image; p=0.044) when compared to WT ones. The number of apoptotic cells was similar in CTGF and WT+ONA optic nerves (p=1.000). No alterations were noted when comparing the number of cleaved caspase 3^+^ cells in CTGF+ONA optic nerves to WT+ONA (p=1.000) as well as CTGF animals (p=0.970; **Figure 6C**).

*Casp3* (caspase 3) mRNA expression levels were analyzed *via* RT-qPCR. The mRNA expression levels of *Casp3* did not differ in WT+ONA (0.83-fold expression; p=0.168), CTGF (1.46-fold expression; p=0.285), and CTGF+ONA optic nerves (0.99-fold expression; p=0.946) compared to WT (**Figure 6D**).

To identify which caspase pathway is involved, the mRNA expression levels of *Casp7* (caspase 7) were investigated. A tendency towards an upregulation was noted in WT+ONA optic nerves (1.74-fold expression; p=0.079). The *Casp7* mRNA expression levels in CTGF mice were slightly increased (3.09-fold expression) but was not significantly different from WT animals (p=0.087). No changes were noted in the *Casp7* mRNA expression levels of the CTGF+ONA optic nerves compared to WT (1.10-fold expression; p=0.606; **Figure 6E**).

The mRNA expression levels of *Casp8* (caspase 8) were examined with RT-qPCR. In WT+ONA optic nerves, the mRNA expression levels of *Casp8* were significantly upregulated (1.77-fold expression; p=0.005). Contrary, the *Casp8* mRNA levels were not altered in CTGF mice compared to WT (1.74-fold expression; p=0.316). A significant upregulation of *Casp8* mRNA expression levels was revealed in CTGF+ONA optic nerves (1.41-fold expression; p=0.003; **Figure 6F**).

*Casp9* (caspase 9) mRNA expression levels were analyzed *via* RT-qPCR. The mRNA expression levels of *Casp9* did not differ in WT+ONA (1.37-fold expression; p=0.241), CTGF (1.58-fold expression; p=0.100), and CTGF+ONA optic nerves (1.09-fold expression; p=0.638) compared to WT (**Figure 6G**).

In summary, a higher number of TUNEL^+^ and cleaved caspase 3^+^ cells was evident in all glaucoma groups. *Casp8* expression levels were upregulated in WT+ONA and CTGF+ONA optic nerves.

### 3.8 Enhanced loss of retinal ganglion cells in CTGF+ONA mice

**Figure 7:**
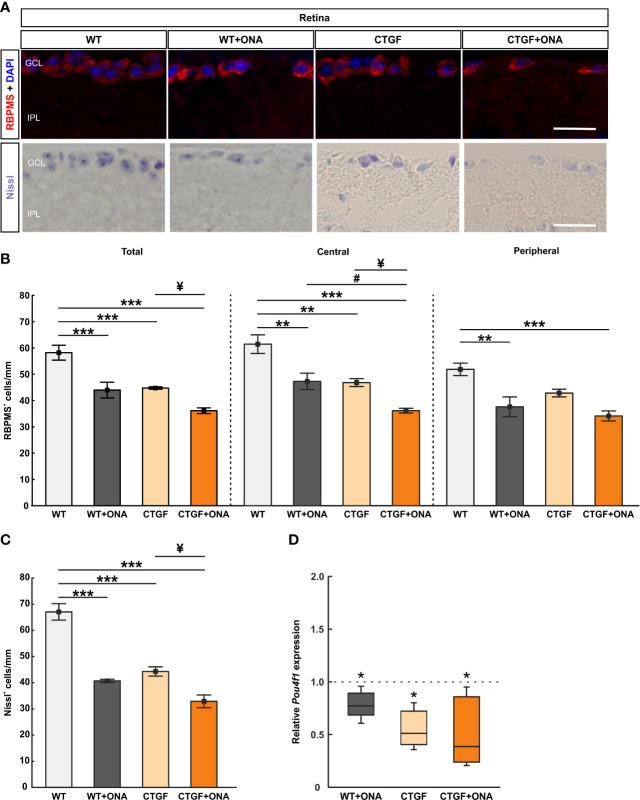
Additive loss of retinal ganglion cells in CTGF+ONA retinae. **(A)** Retinal cross-sections were stained with Nissl to label neurons. Further, an antibody against anti-RBPMS (red) labeled RGCs. DAPI was used to counterstain cell nuclei (blue). **(B)** The total number of RGCs was significantly decreased in WT+ONA, CTGF, and CTGF+ONA retinae compared to WT. Further, RGC counts were significantly lower in CTGF+ONA mice in comparison to CTGF mice. In the central part of the retina, the number of RGCs was diminished in WT+ONA, CTGF, and CTGF+ONA animals compared to WT. In addition, fewer RGCs were counted in CTGF+ONA retinae compared to WT+ONA and CTGF mice. In the peripheral area of the retina, RGCs numbers were lower in WT+ONA and CTGF+ONA mice. **(C)** The number of neurons in the GCL was significantly decreased in WT+ONA, CTGF, and CTGF+ONA retinae compared to WT. While no alterations were noted when comparing WT+ONA to CTGF+ONA mice, significantly lower numbers of neurons were observed in CTGF+ONA animals compared to CTGF ones. **(D)** The mRNA expression levels of *Pou4f1* were significantly downregulated in WT+ONA, CTGF, and CTGF+ONA mice. GCL=ganglion cell layer; IPL=inner plexiform layer. Values are median ± interquartile range ± range for immunohistology and median ± quartile ± minimum/maximum for RT-qPCR. The dotted line in **(D)** represents the relative expression of the WT group. *p<0.05, **p<0.01, and ***p<0.001 *vs*. WT; ^#^p<0.05 *vs*. WT+ONA; ^¥^p<0.05 *vs*. CTGF. Scale bars: 20 µm.

The number of RGCs was evaluated by staining retinal cross-sections with a specific antibody against RBPMS at six weeks (**Figure 7A**). RBPMS^+^ RGC counts were significantly decreased in WT+ONA retinae (43.98 ± 3.00 cells/mm) compared to WT (58.19 ± 2.83 cells/mm; p<0.001). Also, significantly fewer RGCs were noted in CTGF (44.75 ± 0.45 cells/mm; p<0.001) and CTGF+ONA mice (36.14 ± 1.09 cells/mm; p<0.001) in comparison to WT. A trend towards a decrease was observed when comparing CTGF+ONA retinae with the WT+ONA group (p=0.068). Further, the number of RGCs was significantly decreased in the CTGF+ONA group in contrast to CTGF (p=0.039; **Figure 7B**).

Additionally, the number of RGCs was distinguished between central and peripheral areas of the retina. In the central areas, WT+ONA retinae (47.61 ± 3.13 cells/mm) displayed a significant RGC loss compared to WT mice (61.85 ± 3.57 cells/mm; p=0.003). Significantly fewer RGCs in the central region were also noted in CTGF (47.15 ± 1.50 cells/mm; p=0.002) and CTGF+ONA animals (36.39 ± 0.92 cells/mm; p<0.001) compared to the WT group. No changes were noted when comparing the number of RGCs in the central region in WT+ONA and CTGF mice (p=0.999). A significant lower number of RGCs was revealed in CTGF+ONA retinae compared to WT+ONA (p=0.020) and CTGF mice (p=0.027; **Figure 7B**). In the peripheral area of the retina, the number of RGCs was decreased in WT+ONA mice (37.86 ± 3.80 cells/mm) in comparison to WT animals (52.20 ± 2.38 cells/mm; p=0.002). No changes were observed in the CTGF group (43.13 ± 1.46 cells/mm) compared to WT ones (p=0.080). The number of RGCs was significantly diminished in CTGF+ONA mice when compared to WT ones (34.36 ± 1.92 cells/mm; p<0.001). No alterations were observed when comparing WT+ONA and CTGF mice (p=0.473) as well as when comparing CTGF+ONA animals with WT+ONA (p=0.767) and CTGF retinae (p=0.094; **Figure 7B**).

To examine the number of neurons in the GCL a Nissl staining was conducted (**Figure 7A**). In WT+ONA retinae (40.67 ± 0.61 cells/mm) the number of neurons was significantly lower than in WT mice (67.07 ± 3.14 cells/mm; p<0.001). Also, CTGF animals (44.30 ± 1.76 cells/mm) displayed fewer neurons than WT ones (p<0.001). Furthermore, a significantly lower number of neurons was observed in CTGF+ONA retinae (32.91 ± 2.43 cells/mm) when compared to WT mice (p<0.001). No changes were noted between WT+ONA and CTGF+ONA retina sections (p=0.097). Significantly fewer Nissl marked neurons were seen in CTGF+ONA mice compared to CTGF ones (p=0.010; **Figure 7C**).

The mRNA expression levels of *Pou4f1* (Pou class 4 homeobox 1; RGCs) were evaluated *via* RT-qPCR analyses. In accordance with the immunohistology, a significant downregulation could be observed in WT+ONA (0.72-fold expression; p=0.031), CTGF (0.51-fold expression; p=0.036), and CTGF+ONA retinae (0.39-fold expression; p=0.036) compared to WT controls (**Figure 7D**).

Summarized, all glaucoma groups displayed decreased numbers of RGC, with the greatest loss in CTGF+ONA retinae.

### 3.9 More astrogliosis in CTGF and CTGF+ONA retinae

**Figure 8:**
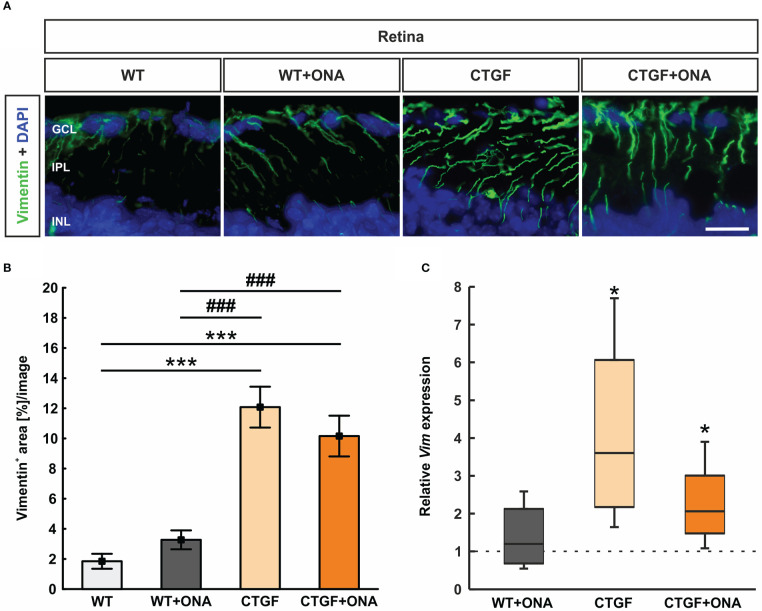
Stronger macrogliosis in CTGF and CTGF+ONA retinae. **(A)** Cross-sections were labelled with an antibody against anti-vimentin (green) to mark astrocytes and DAPI stained cell nuclei (blue). **(B)** The vimentin^+^ area was significantly increased in CTGF and CTGF+ONA retinae compared to WT as well as WT+ONA mice. **(C)** The mRNA expression levels of *Vim* was not changed in WT+ONA retinae, but a significant upregulation was noted in CTGF and CTGF+ONA retinae. GCL=ganglion cell layer; IPL=inner plexiform layer; INL=inner nuclear layer. Values are mean ± SEM for immunohistology and median ± quartile ± minimum/maximum for RT-qPCR. The dotted line in **(C)** represents the relative expression of the WT group. *p<0.05 and ***p<0.001 *vs*. WT; ^###^p<0.001 *vs*. WT+ONA. Scale bar: 20 µm.

Retinal cross-sections were labeled with an antibody against vimentin (Mueller cells; **Figure 8A**). The vimentin staining area in the WT+ONA group (3.27 ± 0.63 [%] area/image) was comparable to WT retinae (1.85 ± 0.49 [%] area/image; p=0.768). A significant increase of the vimentin^+^ area was observed in CTGF (12.08 ± 1.36 [%] area/image; p<0.001) and CTGF+ONA animals (10.16 ± 1.35 [%] area/image; p<0.001) compared to WT mice as well as compared to WT+ONA retina (both: p<0.001). The vimentin area in CTGF+ONA and CTGF mice was very similar (p=0.563; **Figure 8B**).

The mRNA levels of *Vim* were analyzed through RT-qPCR. Consistent with the immunohistological results, the *Vim* mRNA expression levels were not altered in WT+ONA animals (1.20-fold expression; p=0.559). A significant upregulation of *Vim* mRNA levels could be noted in CTGF (3.60-fold expression; p=0.020) as well as in CTGF+ONA retinae (2.06-fold expression; p=0.041; **Figure 8C**).

In summary, an increased macroglia response was only observed in CTGF and CTGF+ONA retinae.

### 3.10 Additive microglia numbers in CTGF+ONA retinae

**Figure 9:**
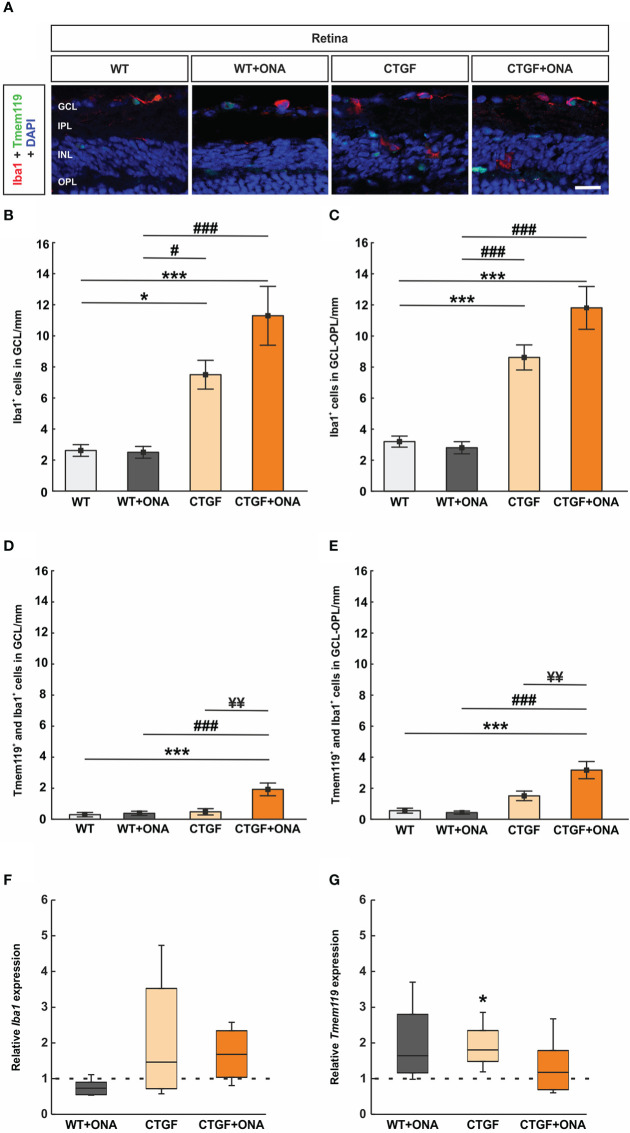
Increased microglia numbers in CTGF+ONA mice. **(A)** Retinal cross-sections were stained for microglia/macrophages with anti-Iba1 (red) and microglia with anti-Iba1 plus anti-Tmem119 (green). DAPI counterstained cell nuclei (blue). **(B)** The number of Iba1^+^ cells in the GCL was not different in the WT+ONA group compared to WT. Significantly more cells were observed in CTGF and CTGF+ONA retinae compared to WT or WT+ONA mice. **(C)** CTGF+ONA retinae displayed significantly more Tmem119^+^ and Iba1^+^ cells in the GCL compared to WT, WT+ONA, and CTGF animals. **(D)** The number of Iba1^+^ cells in the GCL to OPL was not altered in WT+ONA group in comparison to WT. Significantly more cells were observed in CTGF and CTGF+ONA retinae compared to WT or WT+ONA mice. **(E)** Significantly more Tmem119^+^ and Iba1^+^ cells in the GCL to OPL were observed in CTGF+ONA retinae compared to WT, WT+ONA, and CTGF animals. **(F)** No changes were noted in the *Iba1* expression levels within all groups. **(G)** The mRNA expression levels of *Tmem119* were not altered in WT+ONA and CTGF+ONA mice but were significantly upregulated in CTGF retinae compared to WT controls. GCL=ganglion cell layer, IPL=inner plexiform layer, INL=inner nuclear layer, OPL=outer plexiform layer. Values are mean ± SEM for immunohistology and median ± quartile ± minimum/maximum for RT-qPCR. The dotted lines in **(F**, **G)** represent the relative expression of the WT group. *p<0.05 and ***p<0.001 *vs*. WT; ^#^p<0.05 and ^###^p<0.001 *vs*. WT+ONA; ^¥¥^p<0.01 *vs*. CTGF. Scale bars: 20 µm.

As with the optic nerve, anti-Iba1 labeled microglia and macrophages, while anti-Tmem119 plus anti-Iba1 marked microglia. Cells were counted in the GCL alone as well as from the GCL to the ONL (**Figure 9A**). In the GCL, the number of Iba1^+^ cells was comparable in WT+ONA (2.50 ± 0.38 cells/mm) and WT retinae (2.62 ± 0.38 cells/mm; p=1.000). Significantly more Iba1^+^ cells were observed in CTGF retinae (7.50 ± 0.93 cells/mm) compared to WT (p=0.016) and WT+ONA ones (p=0.013). Moreover, a significant increase in Iba1^+^ cell numbers was found in CTGF+ONA mice (11.29 ± 1.89 cells/mm) in comparison to WT and WT+ONA animals (both: p<0.001). The mean Iba1^+^ cell number was about 51% higher in CTGF+ONA retina than in CTGF retinae (p=0.083; **Figure 9B**).

In the GCL to the ONL, the number of Iba1^+^ cells was comparable in WT+ONA (2.80 ± 0.39 cells/mm) and WT retinae (3.20 ± 0.36 cells/mm; p=0.987). Significantly more Iba1^+^ cells were observed in CTGF retinae (8.62 ± 0.81 cells/mm) compared to WT and WT+ONA ones (both: p<0.001). Moreover, a significant increase in Iba1^+^ cell numbers was found in CTGF+ONA retinae (11.81 ± 1.37 cells/mm) compared to WT or WT+ONA animals (both: p<0.001). A trend towards significantly more cells was noted in CTGF+ONA retinae in contrast to CTGF retinae (p=0.051; **Figure 9C**).

The number of Tmem119^+^ and Iba1^+^ cells in the GCL was not altered in WT+ONA (0.38 ± 0.14 cells/mm; p=0.994) and CTGF mice (0.48 ± 0.20 cells/mm; p=0.951) compared to WT animals (0.29 ± 0.44 cells/mm). No difference was observed between CTGF and WT+ONA retinae (p=0.993). In contrast, significantly more Tmem119^+^ and Iba1^+^ cells were noted in CTGF+ONA retinae (1.93 ± 0.41 cells/mm) when compared to WT (p<0.001), WT+ONA (p<0.001), or CTGF animals (p=0.001; **Figure 9D**).

The number of Tmem119^+^ and Iba1^+^ cells in GCL to ONL was not altered in WT+ONA (0.43 ± 0.10 cells/mm; p=0.993) and CTGF mice (1.51 ± 0.31 cells/mm; p=0.192) compared to WT animals (0.56 ± 0.16 cells/mm). Also, counts in CTGF and WT+ONA retinae were alike (p=0.115). In contrast, significantly more Tmem119^+^ and Iba1^+^ cells were noted in CTGF+ONA retinae (3.17 ± 0.55 cells/mm) when compared to WT (p<0.001) as well as WT+ONA (p<0.001) and CTGF animals (p=0.006; **Figure 9E**).

The *Iba1* mRNA expression levels in the retinae were analyzed *via* RT-qPCR. The analyses revealed no differences in the *Iba1* mRNA expression levels in WT+ONA (0.73-fold expression; p=0.065), CTGF (1.46-fold expression; p=0.181), and CTGF+ONA retinae (1.68-fold expression; p=0.112; **Figure 9F**) to WT levels.

Furthermore, *Tmem119* mRNA levels were analyzed. The *Tmem119* mRNA levels remained unchanged in WT+ONA retinae (1.64-fold expression; p=0.081), while a significant upregulation could be noted in CTGF animals (1.81-fold expression; p=0.016) compared to WT. The mRNA expression levels of *Tmem119* were not altered in the CTGF+ONA group (1.78-fold expression; p=0.484; **Figure 9G**).

In summary, the number of Iba1^+^ cells was enhanced in CTGF and CTGF+ONA retinae, while the highest counts were seen in the CTGF+ONA group. Further, only in CTGF+ONA mice, significantly more Tmem119^+^ and Iba1^+^ microglia could be detected.

## 4 Discussion

To prevent vision loss more effectively in patients with glaucoma, new therapeutic options in addition to lowering IOP need to be found. However, due to the complex and multifactorial nature of this disease, it is difficult to understand the underlying pathologies in their entirety. Animal models can serve as a good way to approach this aim. Currently, glaucoma models often focus on only one aspect of the disease, e.g., elevated IOP, glutamate excitotoxity, or immunological alterations (71, 72). In our study we combined for the first time the CTGF high-pressure model with the autoimmune normal-pressure model.

In this study, we immunized six-week-old CTGF and WT mice with ONA, while controls, for each strain, received sodium chloride. Six weeks later, at the age of twelve weeks, we noted a significant IOP elevation in CTGF and CTGF+ONA mice when compared to their respective baseline values. Although the IOP was elevated, this increase was mild. In CTGF mice, previous studies showed no IOP changes in ten-week-old animals, while at 15 weeks of age, a significant IOP elevation was observed (42). In the current work, we examined the mice at twelve weeks of age. Thus, we assume that the IOP in CTGF mice is starting to raise around this age, already leading to glaucomatous damage. Other studies have also shown that a mild IOP elevation can lead to loss and dysfunction of RGCs. In a bead model, in which IOP was raised about 3 mmHg for two weeks, multielectrode assays revealed accelerated temporal properties of RGC photopic responses in the receptive field centers (73). In another OHT study, using injections of hypertonic saline, a mild IOP increase resulted in RGC loss accompanied by complement activation (37). Further, in both individual models, CTGF and ONA immunization, activation of the immune system was observed prior to glaucomatous damage (36, 43, 46). Hence, we suggest that not only the IOP elevation damages RCGs and optic nerves but also the underlying immune responses in both CTGF and in CTGF+ONA mice.

Similar results were gained regarding the retinal function. In our study, a significant loss of a- and b-wave amplitudes was noted only at the highest light intensity of 25 cd*m/s^2^ in CTGF and CTGF+ONA mice. Previously, we detected a decreased b-wave amplitude in 15-week-old CTGF animals (42). In contrast, ONA immunization alone does not appear to affect retinal function. Wiemann et al. observed no alterations in the a- and b-wave amplitudes ten weeks after immunization with ONA (48). Hence, we assume that the decrease in the ERG amplitudes might be attributed to the mechanical damage induced by IOP elevation, leading to an impaired signal transduction. The combination with immune response damage did not lead to a greater loss of retinal function at this point in time. Nonetheless, retinal function in the new model needs to be further examined by using pattern ERGs to monitor RGC function.

### 4.1 Pronounced optic nerve degeneration and RGC loss in CTGF+ONA mice

Optic nerve degeneration and a loss of RGCs are hallmarks of glaucoma disease (2, 74). RGCs transmit the visual stimuli from the retina to the brain through their axons (75, 76). Hence, degeneration of either the cell bodies or the axons leads to impaired transmission and thus to vision loss. We have previously shown that immunization with ONA leads to loss of RGCs and degeneration of the corresponding optic nerves after six weeks (47). When the CTGF mouse was established, the mice had an FVB/NxCD1 background and showed increased IOP and optic nerve axon loss already at four weeks of age (41). Afterwards, mice were backcrossed to a CD1 background to obtain a strain more susceptible to IOP alterations (77, 78). With this background, we observed RGC degeneration at 15 weeks of age (42). As mentioned above, in the study presented here, evaluations were performed six weeks after immunization, thus in twelve-week-old mice. Here, a more severe damage could be shown especially in the optic nerve structure and RGCs of CTGF+ONA mice. In the optic nerve, more cell infiltration was noted in WT+ONA and CTGF optic nerves, but the highest cell infiltration score was observed in CTGF+ONA mice. Further, disruption of myelin sheaths as well as significant downregulation of *Mbp* were visible only in CTGF+ONA optic nerves. However, not only was the optic nerve more severely damaged, the loss of RGCs was also more prominent in CTGF+ONA mice. This suggests that the combination of two risk factors enhanced the harm to the optic nerve and RGC bodies.

Interestingly, although mRNA expression levels of *Olig2* were downregulated in all three glaucoma groups, the number of oligodendrocytes was lower only in WT+ONA optic nerves. A significant lower number of oligodendrocytes was also observed ten weeks after ONA immunization (48). In contrast, CTGF itself seems to interplay with Olig2. Among CTGF proteins, integrins, the low-density lipoprotein receptor-related protein 1 and the vascular endothelial growth factor (VEGF) are known to modulate the oligodendrocyte development in several aspects (79–81). While VEGF and ανβ5 integrin are involved in the migration of oligodendrocyte precursors, ανβ3 integrin induced their proliferation (82–84). By applying exogenous CTGF in the ventricles of neonatal rodent brains, a reduction of mature oligodendrocytes was noted (85). Yu et al. generated a CTGF knock-out mice, where the CTGF protein expression is eliminated only in excitatory neurons in the forebrain. Depending on the localization, the CTGF knock-out showed either an increased or an unchanged number of mature oligodendrocytes compared to controls (86). The authors suggest that CTGF is secreted from the soma or dendrites of subplate neurons and suppresses the oligodendrocyte maturation in a paracrine manner. In our model, oligodendrocytes in CTGF animals seem to be more resistant at this time point. This should be analyzed more precisely in further studies.

### 4.2 Optic nerve apoptosis in all glaucoma optic nerves

Cell death mechanisms, such as apoptosis, play a major role in glaucomatous degeneration in patients and are also a focus of investigation in animal models (50, 87–89). Since little is currently known about apoptotic mechanisms in WT+ONA, CTGF, or CTGF+ONA optic nerves, we examined the number of TUNEL^+^ cells as well as cleaved caspase 3^+^ cells and corresponding gene levels. The number of apoptotic cells was significantly increased in all glaucoma groups compared to WT, but no additive effect was seen in CTGF+ONA optic nerves. Caspase 3 is an effector caspase, which degrades the cell compartments to induce morphological changes for apoptosis (90). It is possible that apoptotic mechanisms occurred earlier in the time course of the CTGF+ONA model. We further analyzed the mRNA expression levels of *Casp7*, *Casp8*, and *Casp9*. We noted a significant upregulation of *Casp8* mRNA levels in WT+ONA and CTGF+ONA retinae. Caspase 8 is an initiator caspase in death receptor-induced signaling of apoptosis (91, 92). In the EAG model, we could previously detect more caspase 8^+^ cells in the GCL of immunized rats (93). Hence, the extrinsic pathway seems to be activated in the IOP-independent model. This is consistent with the findings of current findings since only WT+ONA and CTGF+ONA animals displayed elevated *Casp8* levels. No alterations were observed for *Casp7* and *Casp9*, suggesting that the intrinsic pathway plays a minor role. Future studies should address the role of apoptotic processes in more detail in this new combination model. Further, other cell death mechanisms, like autophagy, also might be involved. In the last years, autophagic processes were described as a contributor to the cell degeneration in glaucoma (53, 93–95).

### 4.3 Microglia activation predominantly in CTGF+ONA retinae

Microglia play a crucial role in innate immunity and in neurodegenerative disorders. In the developing retina, they are involved in angiogenesis and neural circuits. On the other hand, in the mature retina, microglia regulate retinal neuron activity as well as synaptic integrity (96–98). After being exposed to pathological stimuli they respond quickly and can migrate to the site of injury within 24 hours (99). In the optic nerve head of human glaucoma donors, Yuan and Neufeld discovered that microglia expressed different cytokines, such as tumor necrosis factor-α or transforming growth factor-β2 (100). In glaucoma disease, microglia activation is considered as one of the earliest events. In the autoimmune glaucoma model, microglia activation was observed even before a RGC loss occurred (46). Also, in the ocular hypertension (OHT) DBA2/J mouse, activation and redistribution of microglia were noted prior to RGC degeneration (101). In our study, the number of optic nerve macrophages/microglia did not differ within the groups, only the *Iba1* mRNA expression levels were upregulated in CTGF+ONA optic nerves. As noted for optic nerve apoptosis, the examined point in time could be too late to register microglia activation, since the degeneration of the tissue is already quite severe.

In contrast, the number of Iba1^+^ macroglia/macrophages and Tmem119^+^ and Iba1^+^ microglia in the retina were significantly increased in CTGF and CTGF+ONA mice, while no changes were noted in WT+ONA animals. The highest number of cells was observed in the CTGF+ONA group. Although all glaucoma groups revealed a loss of RGCs, the damage to the retina was not that severe compared to the optic nerve degeneration. This might be the reason why in contrast to the optic nerves, a microglia activation was still evident in the retinae. It seems odd that the number of macrophages and microglia was not altered in WT+ONA retinae, since this was shown in a previous study (47). However, this study was performed in another mouse strain (129S2/SvPasCrl). It is known that microglia response is strain dependent. For example, the number of Iba1^+^ cells was lowest in the FVB and DBA/2 strain (102). Interestingly, the combination of CTGF mice and ONA immunization triggered an enhanced microglia activation in the retina. In human retinae, distinct and specific staining of CTGF was observed in paravascular microglia, while in diabetic retinae CTGF immunostaining shifted towards pericytes (103). On the other hand, a study by Abu El-Asrat et al. observed immunoreactivity of CTGF in RGCs and microglia of diabetic patients (104). In a forebrain specific CTGF knock-out model, seizures were induced by pentylenetetrazole. The knock-out mice did not display alterations in microglia number, heterogeneity, or shape (105). All these results lead to the assumption that the overexpression of CTGF in combination with an immunization increased the microglia response in the model.

### 4.4 Enhanced macrogliosis in CTGF+ONA optic nerves

In the optic nerve head, astroglia are the most abundant cells and in the nervous system, S100B is mainly expressed by astrocytes (106). After stimulation or injury, they become reactive and express GFAP (107). In glaucoma it is evident that astrocytes in the optic nerve head rearrange their actin cytoskeleton coinciding with astrocyte activity and extracellular matrix depositions (108). In our study, all glaucoma groups displayed disarranged GFAP^+^ and S100B^+^ astroglia in their optic nerves. After ONA immunization, an enhanced macrogliosis was already shown in previous studies (48, 109). For CTGF mice, macroglia were not investigated in the optic nerves by now. In this study, the disarrangement of GFAP and S100B was similar with WT+ONA mice. However, by combining these two models, the loss of structural integrity was most severe in CTGF+ONA optic nerves and in addition, a significant upregulation of *Gfap* mRNA levels was revealed solely in this group. Studies showed that in response to an elevated IOP, astrocytes and upregulated cytoskeletal proteins tightly surround the optic nerve head. After long-term IOP enhancement, astrocytes then decrease and can even disappear (110). Further, reactive astrocytes mediate different inflammatory pathways, such as tumor necrosis factor α or inflammasome-associated regulators, as shown in an OHT rat model (111). Moreover, astrocytes in the optic nerve head seem to engulf axons and promote their degeneration (112, 113).

In the retina, Müller cells span through all retinal layers and are involved in glucose metabolism, retinal blood flow regulation, neurotransmitter transmission, as well as balance of homoeostasis (114). In pathological situations, Müller cells can regulate immunity and phagocytic cells and undergo gliosis (115). In human glaucoma donor eyes, Müller cells and astrocytes displayed a hypertrophic morphology and increased GFAP immunostaining (116). In the CTGF mice at 15 weeks of age, an increase of the glutamine synthetase^+^ area, but not of the GFAP^+^ or vimentin^+^ area could be noted (42). In the autoimmune glaucoma model, an enhancement of macroglia in the retina depends on the point in time. Six weeks after ONA immunization, the GFAP^+^ area did not differ from control animals (47). Later, ten weeks after immunization, an increase of the GFAP^+^ immunostaining in ONA mice could be observed (48). We suggest that the retinal damage is not that severe for macroglia to switch to an active state six weeks after immunization. This is in accordance with the results of our study presented here. An enhancement of the vimentin^+^ area was only significantly higher in CTGF and CTGF+ONA retinae, suggesting that the mechanisms in the CTGF mouse strain are responsible for this increase.

## 5 Conclusions

The main challenge in developing therapeutic approaches for glaucoma is the complexity of the disease. An elevated IOP and the subsequent mechanical damage play a major role but are not the only contributing factor. By combining two risk factors in one *in vivo* model, we have now been able to explore the multifactorial genesis more precisely. In this new CTGF+ONA model, a more pronounced damage of the optic nerve and the RGCs was observed. The apoptotic processes in the optic nerves revealed a contribution of the extrinsic pathway. In future studies, this model may help to gain further insights into the pathomechanisms and could lead to new leverage points for therapies.

## Data availability statement

The original contributions presented in the study are included in the article/**Supplementary Material**. Further inquiries can be directed to the corresponding author.

## Ethics statement

The animal study was reviewed and approved by Landesamt für Natur, Umwelt und Verbraucherschutz.

## Author contributions

SR performed experiments, analyzed data, and wrote the manuscript; RG, KS, JT, and MA performed experiments and analyzed data. RF and BD revised the manuscript. SJ conceived the study and revised the manuscript. All authors contributed to the article and approved the submitted version.
