# Supplementary material for: Enhanced glaucomatous damage accompanied by glial response in a new multifactorial mouse model
Source: Front Immunol. 2023 Jan 17;13:1017076. doi: 10.3389/fimmu.2022.1017076 (PMC9887307; doi:10.3389/fimmu.2022.1017076)
Supplement: Supplementary file 1 [file DataSheet_1.pdf]

Supplementary Material

Supplementary Figures

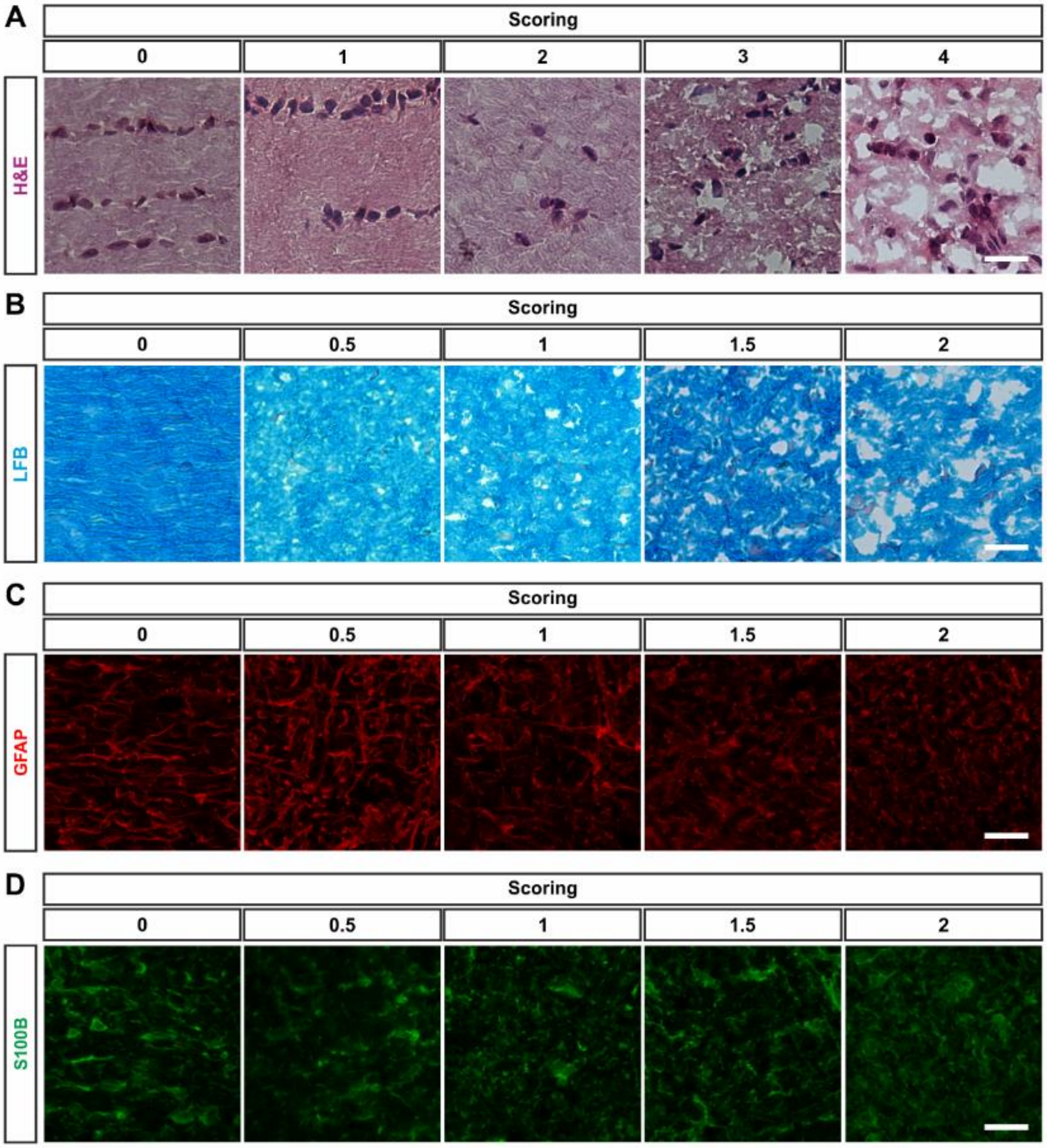

Supplementary Figure 1: Exemplary images of the different optic nerve scores. **(A)** The H&E images were scored from 0=no infiltration, 1=mild cellular infiltration, 2=moderate infiltration, 3=severe infiltration, to 4=massive infiltration with formation of cellular conglomerates. **(B)** LFB images were assessed with following score: 0=no demyelination, 0.5=mild demyelination, 1=moderate demyelination, 1.5=advanced demyelination, and 2=severe demyelination up to complete loss of structural integrity, **(C)** GFAP$^+$ and **(D)** S100B$^+$ astrocytes were graded as followed: 0=astrocyte structure intact, 0.5=mild astrocyte structure loss, 1=moderate astrocyte structure loss, 1.5=advanced structure loss, and 2=severe up to complete loss of astrocyte structural integrity. Scale bars: 20 µm.

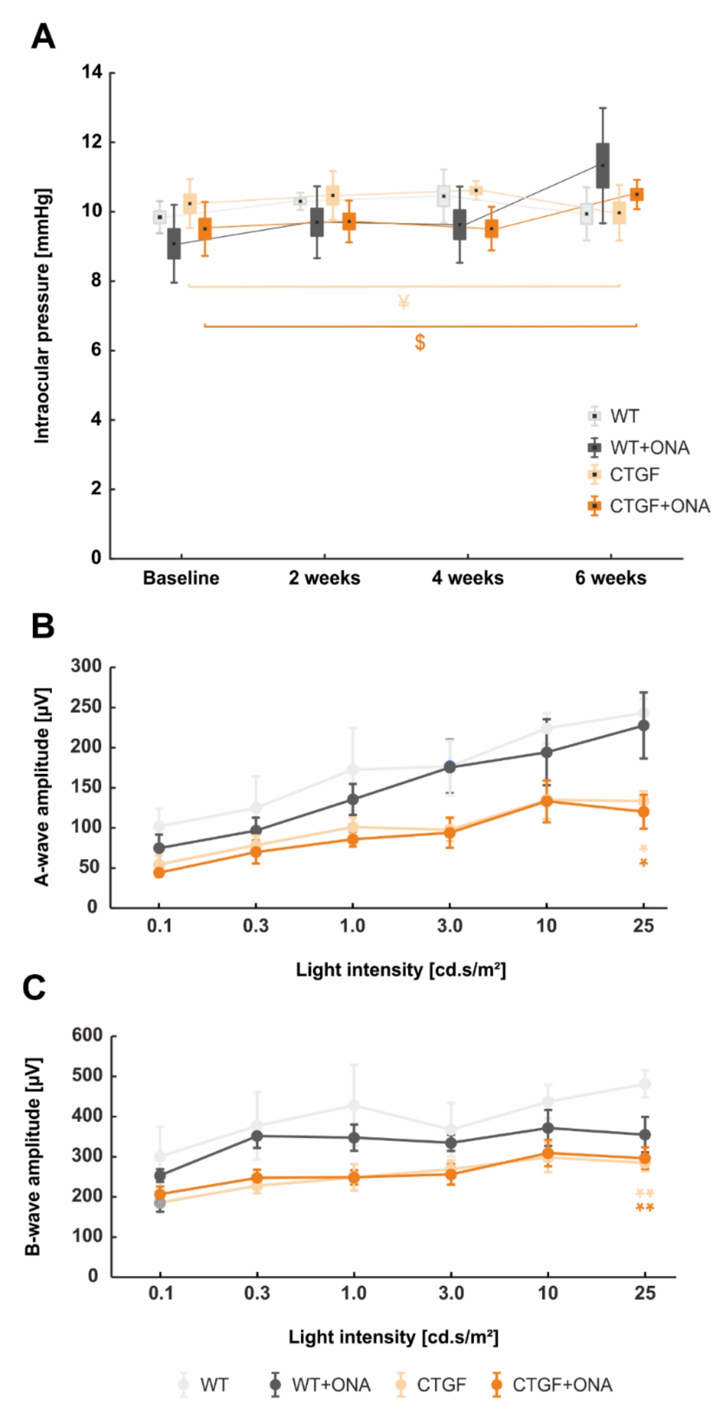

Supplementary Figure 2: (A) IOP was measured before (baseline) as well as two, four, and six weeks after immunization. The IOP did not alter within the points in time. However, the IOP of the CTGF group was significantly higher at six weeks compared to CTGF animals at baseline. Additionally, a significant higher IOP could be noted in CTGF+ONA mice at six weeks compared to baseline. **(B)** Six weeks after immunization, the a-wave amplitudes revealed a significant loss only at a light intensity of 25 cd*m/s^2 in CTGF as well as in CTGF+ONA animals. **(C)** Also, a significant lower b-wave amplitude was revealed in CTGF and CTGF+ONA mice at 25 cd*m/s^2. *$p<0.05$ and **$p<0.01$ vs. WT; $^{¥}p<0.05$ vs. CTGF; $^{\$}p<0.05$ vs. CTGF+ONA.
